# Rationale and design of a randomized controlled trial of directly observed hepatitis C treatment delivered in methadone clinics

**DOI:** 10.1186/1471-2334-11-315

**Published:** 2011-11-12

**Authors:** Alain H Litwin, Karina M Berg, Xuan Li, Jennifer Hidalgo, Julia H Arnsten

**Affiliations:** 1 Division of General Internal Medicine, Department of Medicine, Albert Einstein College of Medicine and Montefiore Medical Center, 111 East 210th Street, Bronx, New York, 10467, USA; 2 Division of Substance Abuse, Department of Psychiatry and Behavioral Sciences, Albert Einstein College of Medicine and Montefiore Medical Center, 111 East 210th Street, Bronx, New York, 10467, USA; 3 Department of Epidemiology and Population Health, Albert Einstein College of Medicine and Montefiore Medical Center, 111 East 210th Street, Bronx, New York, 10467, USA

## Abstract

**Background:**

Most methadone-maintained injection drug users (IDUs) have been infected with hepatitis C virus (HCV), but few initiate HCV treatment. Physicians may be reluctant to treat HCV in IDUs because of concerns about treatment adherence, psychiatric comorbidity, or ongoing drug use. Optimal HCV management approaches for IDUs remain unknown. We are conducting a randomized controlled trial in a network of nine methadone clinics with onsite HCV care to determine whether modified directly observed therapy (mDOT), compared to treatment as usual (TAU), improves adherence and virologic outcomes among opioid users.

**Methods/Design:**

We plan to enroll 80 HCV-infected adults initiating care with pegylated interferon alfa-2a (IFN) plus ribavirin, and randomize them to mDOT (directly observed daily ribavirin plus provider-administered weekly IFN) or TAU (self-administered ribavirin plus provider-administered weekly IFN). Our outcome measures are: 1) self-reported and pill count adherence, and 2) end of treatment response (ETR) or sustained viral response (SVR). We will use mixed effects linear models to assess differences in pill count adherence between treatment arms (mDOT v. TAU), and we will assess differences between treatment arms in the proportion of subjects with ETR or SVR with chi square tests. Of the first 40 subjects enrolled: 21 have been randomized to mDOT and 19 to TAU. To date, the sample is 77% Latino, 60% HCV genotype-1, 38% active drug users, and 27% HIV-infected. Our overall retention rate at 24 weeks is 92%, 93% in the mDOT arm and 92% in the TAU arm.

**Discussion:**

This paper describes the design and rationale of a randomized clinical trial comparing modified directly observed HCV therapy delivered in a methadone program to on-site treatment as usual. Our trial will allow rigorous evaluation of the efficacy of directly observed HCV therapy (both pegylated interferon and ribavirin) for improving adherence and clinical outcomes. This detailed description of trial methodology can serve as a template for the development of future DOT programs, and can also guide protocols for studies among HCV-infected drug users receiving methadone for opiate dependence.

**Trial Registration:**

ClinicalTrials.gov: NCT01442311

## Background

Drug users account for a disproportionately large burden of hepatitis C (HCV) infection and have been shown in some studies to achieve HCV treatment success rates that are equivalent to non drug users [1]. However, HCV treatment adherence rates in drug users may be suboptimal in patients who use drugs regularly during HCV treatment [2,3]. Because HCV treatment is most effective when patients adhere to at least 80% of the prescribed treatment regimen [4], interventions to improve HCV treatment adherence need to be developed and evaluated among drug users.

Prior research has shown that treatment adherence for other infectious diseases, such as tuberculosis and HIV, is improved among drug users when directly observed therapy (DOT) is administered at methadone maintenance treatment programs [5,6]. HCV treatment may be well suited for DOT because it has a discrete course lasting 24 to 48 weeks, and the backbone of therapy, subcutaneous pegylated interferon injections, is administered weekly rather than daily. Since methadone-maintained drug users attend opiate agonist treatment programs up to six days per week, it is feasible to observe oral ingestion of daily ribavirin, to which adherence may be particularly important for achieving sustained viral response [7-9]. However, DOT for HCV has only recently been evaluated [10,11], and, to our knowledge, no randomized trial has evaluated both directly observed pegylated interferon and directly observed ribavirin administered on-site in a methadone clinic.

We designed the HCV DOT trial to test the efficacy of modified directly observed HCV therapy provided on-site at a methadone clinic. We implemented this randomized trial in a network of methadone clinics that offers on-site HCV treatment by primary care providers, including standard weekly provider-administered pegylated interferon injections [12-14]. The primary objective of our trial is to determine whether modified DOT (mDOT) with both pegylated interferon alfa-2a plus ribavirin is more efficacious for increasing adherence and improving HCV treatment outcomes than treatment as usual (TAU), which includes weekly provider-administered pegylated interferon and self-administered ribavirin. We here provide a detailed methodological description of this trial, which can serve as a template for other investigators designing HCV DOT trials. In addition, we discuss pertinent safety and logistical issues to guide future studies among HCV-infected drug users receiving methadone for opioid dependence. Finally, we report baseline characteristics of our study population.

## Methods/design

### Study setting

The HCV DOT trial is being conducted in the Division of Substance Abuse (DoSA), a network of nine methadone maintenance clinics administered by the Albert Einstein College of Medicine in the Bronx, New York. Together, these affiliated clinics provide care for approximately 3,500 opioid dependent patients, of whom approximately 65% have been infected with HCV and over 50% have chronic hepatitis C. These nine clinics are the sites for study recruitment, delivery of the HCV DOT intervention, and all research visits.

Substance abuse treatment at each DoSA clinic is delivered by a multidisciplinary staff comprised of substance abuse counselors, nurses, a part-time social worker, a part-time psychiatrist, and a medical team consisting of a physician and at least one physician assistant. The medical team provides comprehensive primary medical care, including HCV and HIV care.

### On-site HCV Treatment Program

HCV-infected patients with appropriate insurance (including Medicaid) are offered on-site HCV evaluation and treatment by primary care providers, and are free to choose whether to receive HCV and other medical care at the methadone clinic or elsewhere. Primary care providers receive ongoing HCV-related training from an internist with expertise in providing HCV treatment to drug users, follow standardized HCV treatment protocols [13,15], and have access to expert backup from a hepatologist. Liver biopsies are available at an off-site affiliated medical center. On-site HCV support groups are available at all nine clinics, and are facilitated by a HCV-infected peer and/or clinic staff [12].

Prior to this trial, most patients received provider-administered pegylated interferon alfa-2a injections (180 mcg weekly), and self-administered twice-daily ribavirin. Ribavirin is taken in three different dosages based on HCV genotype, HIV status, and weight: either 400 mg twice daily, 600 mg in the morning and 400 mg in the evening, or 600 mg twice daily. Among trial participants, those in the TAU arm continue to receive standard treatment (once weekly provider-administered pegylated interferon alfa-2a injections) plus self-administered ribavirin.

Our HCV evaluation and treatment protocol (AASLD 2009) requires assessing HCV viral loads at the following time points: baseline (prior to HCV treatment initiation), after HCV treatment initiation (4 weeks, 12 weeks, 24 weeks, and at the end of treatment) [15], and 24 weeks after HCV treatment completion or discontinuation. The planned duration of treatment is 48 weeks for genotype-1 or -4 monoinfected patients and all HIV/HCV coinfected patients, and 24 weeks for genotype-2 or -3 monoinfected patients. Quantitative HCV viral load is performed by Bayer assay which quantifies viral load between 615-7,700,000 IUs/ml. HCV genotype is assessed prior to treatment initiation. DoSA conducts annual HIV testing for all patients (unless they opt out) who are not already known to be HIV-infected. In addition, patients initiating on-site HCV treatment undergo HIV tests as part of our standard treatment protocol.

#### Trial inclusion and exclusion criteria

Potential trial subjects are eligible for inclusion if they are HCV-infected; receive HCV medical care at the methadone clinic; plan to initiate HCV treatment on-site at the methadone clinic within the next three months; are psychiatrically stable as determined by the HCV treatment provider and/or DoSA on-site psychiatrist; attend their DoSA methadone clinic between three and six days per week to receive methadone; and have been on a stable dose of methadone for two weeks prior to the baseline visit. Participants are excluded if they are unable or unwilling to provide informed consent, are currently receiving HCV treatment (pegylated interferon and ribavirin), or if their primary HCV care provider does not agree to their participation in the trial. Participants are also excluded (and referred immediately to the clinic's medical provider) if they report suicidal ideation during the baseline interview.

### Approvals and data safety and monitoring

The trial was approved by the Committee on Clinical Investigations of the Albert Einstein College of Medicine and the Institutional Review Board of Montefiore Medical Center. All participants provide written informed consent. We are testing a low-risk behavioral intervention that is highly integrated with usual clinical care; for this reason we did not create an independent data safety and monitoring board. Instead we established a data safety and monitoring plan, which requires interim analyses after every twenty patients are enrolled to determine whether there are sufficient risks or benefits, or whether significant differences in virological outcomes between study arms have developed that warrant trial cessation.

### Recruitment

Medical providers are asked to invite all patients initiating on-site HCV treatment to consider enrolling in the trial. Subjects also self-refer after seeing flyers posted in clinics, or are referred by other subjects or through HCV support groups. The initial steps in subject recruitment include a brief screening survey, informed consent, and in depth verification of eligibility using medical records and discussion with providers.

### Randomization

Random-number tables are used to allocate subjects to the mDOT intervention arm or the TAU control arm. Randomization is stratified by both HIV status and by HCV genotype, because of the influence of these two factors on virologic outcome. Four distinct randomization lists were created: (1) HIV+, genotype 1 or 4, (2) HIV+, genotype 2 or 3, (3) HIV-, genotype 1 or 4, and (4) HIV-, genotype 2 or 3. To ensure comparison groups of roughly equal size, we randomized by blocks within each of these four strata. In addition, blocks were of variable number to minimize the chance of upcoming group assignment being anticipated by research assistants.

### Baseline assessment

The baseline assessment consists of an interview, administered using Audio Computer-Assisted Self-Interview (ACASI) technology to reduce social desirability bias. During the interview the subject simultaneously reads the question on the computer screen and listens to it read aloud through headphones. Baseline HCV viral load, genotype, and results of urine toxicology tests are obtained by chart review. All subjects have urine toxicology testing at least monthly, as this is a requirement for methadone maintenance treatment programs. The baseline interview is conducted prior to HCV treatment initiation, and at this visit all subjects are invited to attend HCV support groups. Following completion of the baseline assessment, participants receive their assignment to either the mDOT or the TAU arm.

### Modified DOT intervention arm (mDOT)

Subjects randomized to the mDOT arm receive weekly provider-administered pegylated interferon alfa-2a injections plus modified directly observed ribavirin therapy. We describe this as modified because ribavirin ingestion is observed at the methadone window three to six days per week based on the participants' methadone pick-up schedule, and only one of two daily doses of ribavirin is observed. Our mDOT intervention has 3 additional components: 1) individually packaged take-home ribavirin doses; 2) nurses check in with subjects regarding side effects; and 3) nurses contact on-site medical providers if they note any adherence problems, refusal of ribavirin, or medication side effects.

Preparing pill trays for use in the mDOT intervention requires communication between the trial's Medical Director, research staff, HCV treatment providers, nurses, community pharmacies, and centralized DoSA pharmacists. For each participant, the Medical Director verifies the ribavirin dose by speaking directly with the HCV provider, and calls in prescriptions to a single designated community pharmacy that delivers medications directly to the DoSA central pharmacy. HCV providers are asked not to write ribavirin prescriptions for mDOT participants for the 24-48 week HCV treatment period, and to contact the Medical Director directly if a participant changes dose or discontinues ribavirin.

To minimize nursing burden, all DOT medications are prepared in advance in individualized pill trays. Pill trays are labeled by research assistants and filled by centralized DoSA pharmacists. Each pill tray holds seven removable single dose pillboxes that contain ribavirin pills. Since ribavirin is prescribed twice daily, we utilize two pill trays: one containing the morning doses (usually 2-3 capsules) and one containing the evening doses (usually 2-3 capsules). Depending on methadone pick-up schedule and dosing frequency, certain doses cannot be observed (i.e., weekend doses, evening doses, and doses to be taken on a non-clinic day). In these instances, participants are given single dose pillboxes, or "take home doses," for each unobserved dose, and are asked to return the pillbox to the nurses at the next clinic visit, whether or not they have taken the pills. Pill trays are delivered from the centralized DoSA pharmacy to the individual methadone clinics every two weeks, along with scheduled methadone deliveries. Nurses instruct the subjects to eat food soon after ingestion of ribavirin, and snacks are available at the nursing station.

### Treatment as usual control arm (TAU)

Subjects randomized to the TAU arm receive standard on-site treatment (weekly provider-administered pegylated interferon alfa-2a injections) and self-administered twice-daily oral ribavirin. Subjects in the TAU arm are dispensed monthly medication bottles of ribavirin, and ingest the ribavirin at home. Since up to 6 tabs of ribavirin (200 mg each) are taken daily (168 tabs monthly), a monthly supply can not fit in a standard medication bottle. Therefore, when the medication is prescribed, the trial's Medical Director contacts the community pharmacy and requests that a subject's monthly medication be packaged in a single large medication bottle to facilitate assessment of a monthly pill count (described below).

### Visit schedule and measures

**Figure 1:**
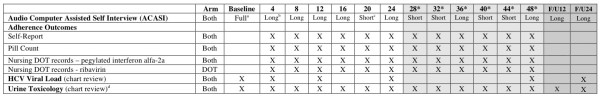
**Schedule of research visits**. * Subjects treated and followed for 48 weeks only (HCV monoinfected - G1/4; HIV/HCV coinfected - all genotypes). a) Full Baseline ACASI survey includes measures of: adherence [33]; opiate withdrawal [34]; recent drug and alcohol use [5,35]; alcohol dependence [36]; HCV-related adherence knowledge [5,37]; attitudes towards HCV medications [5,37]; adherence self-efficacy [5,37]; medication side effects [5]; coping [38]; depression (BDI-II and PHQ-9)[39-41]; psychological distress (hostility subscale)[42-44]; loneliness [45]; social support [46]; acculturation [47,48]; patient-provider relationship [49]; cognitive function [50-52]; Barriers and Facilitators to Care [37]. b) Long ACASI survey includes measures of: adherence [33]; opiate withdrawal [34]; on-site social support; depression (BDI-II and PHQ-9)[39-41]; psychological distress (hostility subscale)[42-44]; recent drug and alcohol use [5,35]; medication side effects [5]; HCV-related adherence knowledge (wks 8 and 16 only) [5,37]; attitudes towards HCV medications (wks 8 and 16 only)[5,37]; adherence self-efficacy (weeks 8 and 16 only) [5,37]. c) Short ACASI survey includes measures of: adherence [33]; depression (BDI-II and PHQ-9)[39-41]; medication side effects [5]; opiate withdrawal [34]. d) Urine tested for methadone, opiates, cocaine, benzodiazepines, barbiturates, and amphetamines.

Frequency and length of research visits during the 24 to 48 week intervention period are the same for subjects in both arms (Figure 1). We standardized the visit schedule to control for any improvement in adherence that might result from trial participation alone. Participants are reimbursed $25 for the baseline visit and for long research visits, and $10 for brief research visits.

### Adherence measures

We use several measures to assess ribavirin adherence: self-report, pill count, and nursing records of directly observed doses and returned pill boxes. Ribavirin pill counts are performed of unconsumed pills in the pill trays in the mDOT arm and in the ribavirin bottle in the TAU arm. We assess adherence to pegylated interferon alfa-2a injections through provider records of directly administered injections.

### Virologic outcomes

We review medical charts to obtain results for HCV viral load tests performed at 4 weeks (rapid viral response), 12 weeks (early viral response), 24 weeks and/or 48 weeks (end of treatment response), and 24 weeks after treatment discontinuation or completion (sustained viral response).

### Psychosocial measures

Psychosocial domains and instruments are listed in Figure 1. The full ACASI interview takes between two and four hours to complete, and is administered at baseline. An abbreviated ACASI interview is administered at all other research visits. Two follow-up research visits are conducted 12 weeks and 24 weeks after treatment completion or discontinuation.

### Specific safety protocols

We utilize several safety protocols to ensure appropriate handling of psychological distress among research subjects, provide timely feedback of HCV treatment related side effects to providers, and ensure safe and accurate administration of ribavirin doses to subjects enrolled in the mDOT arm.

### Psychological Distress

Irritability and depression are the most common neuropsychiatric side effects of HCV treatment [16-18]. At all research visits, either the full Brief Symptom Inventory (BSI) or the BSI Hostility Sub-scale are administered to measure overall psychological distress and irritability/hostility [19]. The Beck Depression Inventory-II (BDI-II), and Patient Health Questionnaire-9 (PHQ-9) are also administered to measure depressive symptoms [20,21]. Research staff inform providers immediately if subjects have suicidal or homicidal thoughts, or have severe depressive symptoms on these screening instruments. Copies of BDI-II and PHQ-9 results are given to providers, as this may facilitate early identification of depression and/or appropriate referrals to psychiatric care.

### Treatment-related side effects

A treatment side effect scale (which measures the degree of 25 HCV treatment-related side effects) is administered at each monthly visit, and the results are given to medical providers. Side effects include fatigue, headache, fever, myalgias/arthlagias, insomnia, nausea, anorexia, alopecia, rash and other common side effects.

### Ribavirin stockpiles and dose changes

We maintain a ribavirin stockpile in our central pharmacy in case there are lapses in subjects' insurance coverage, or delays in delivery of ribavirin from community pharmacies. A stockpile is also stored in individual clinics to accommodate immediate ribavirin doses changes.

### Sample size and power calculations

Sample size calculations were based on our primary outcome of adherence. Effect sizes and other assumptions were based on our prior study of directly observed treatment to antiviral HIV treatment in the same setting, and on a previous HCV treatment study [2,21-23]. We will need 29 subjects in each arm to have 85% power to show at least 14% difference in adherence between trial arms, and 33 subjects in each arm to have 90% power to show a 14% difference. Using the Fisher's exact test, a sample size of 36 per arm will achieve 83% power to detect 31% difference in the percentage of subjects with at least 80% adherence.

### Planned statistical analyses

We hypothesize that adherence to ribavirin (measured by pill count) in the mDOT arm will be higher than adherence in the TAU arm, and that the mDOT arm will have a higher percentage of subjects with at least 80% adherence to ribavirin than the TAU arm. Pill count adherence will be treated as a continuous estimate and will be analyzed using linear mixed-effects models to compare mean pill counts between study arms over the course of the trial.

We will also calculate overall ribavirin exposure, which will take into account three factors: percent of prescribed doses taken (adherence), length of time on treatment (persistence), and proportion of the original dose taken (dose reduction) [24]. Ribavirin adherence and exposure will be calculated for each subject during the first 12 weeks, and during the entire 24- or 48-week treatment course (for those who achieve an EVR).

All analyses will be repeated using self-reported adherence, which has been demonstrated to be a valid measure of adherence to HCV therapy [25]. To account for inherent social desirability biases in self-reported adherence, for the majority of these analyses we will dichotomize this variable as 100% vs. <100% [26]. A logistic mixed-effects model will be used to analyze repeatedly measured self-reported adherence.

### Study sample characteristics

Screening and baseline interviews began in November 30, 2007, and are ongoing. We present the baseline characteristics for the first 40 subjects who have enrolled and initiated on-site HCV treatment through June, 2010): Of these, 21 were randomized to the mDOT arm and 19 to the TAU arm.

**Figure 2:**
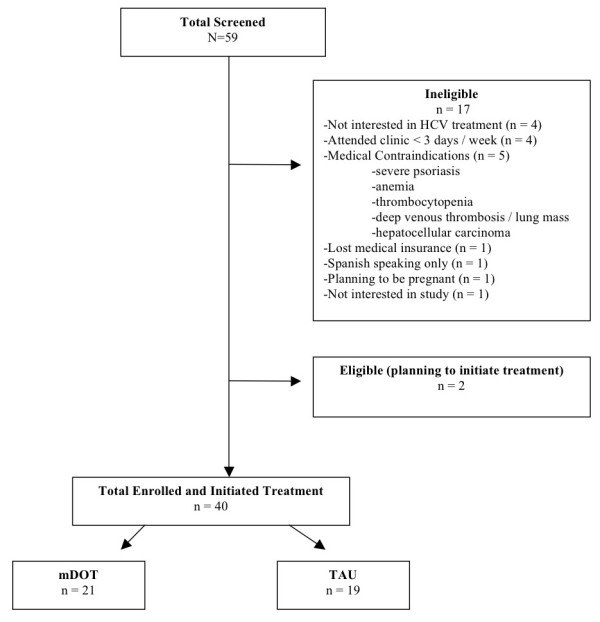
**Flow chart of study recruitment and enrollment**.

To date, a total of 59 subjects have been screened (Figure 2). The overall retention rate during the intervention period is 92%. By arm, retention rates are 93% for DOT and 92% for TAU.

The sample is 55% male, 77% Hispanic, and 15% Black, with a mean age of 48. (Table 1). Thirty-eight per cent self-reported substance use (heroin/cocaine/crack) in the 30 days prior to the baseline interview. The median dose of methadone is 90 mg (IQR 60 - 140). At baseline, 65% of the sample had current comorbid psychiatric conditions: depression, anxiety disorder, psychotic disorders, post-traumatic stress disorder, and/or bipolar disorder.

**Table 1:** Baseline characteristics of study sample (n = 40)

Sociodemograpic	
Age, mean (sd)	48 (6.4)
Gender, n (%)	
Male	22 (55)
Female	18 (45)
Race, n (%)^a^	
White	15 (39)
Black	6 (15)
Other	18 (46)
Ethnicity, n (%)^a^	
Hispanic	30 (77)
Non-Hispanic	9 (23)
Education, n (%)	
Grade 12/GED or lower	37 (93)
College (partial or completed)	3 (7)
Marriage Status, n (%)	
Married/living with partner	20 (50)
Widowed/separated/divorced/single	20 (50)
Employed, n (%)^a^	
Employed	4 (11)
Unable to work/unemployed/other	34 (89)
Housing status, n (%)^a^	
Stable	31 (82)
Unstable	7 (18)
**Psychiatric**	
Psychiatric illness, n (%)^b^	
Current psychiatric illness	26 (65)
No current psychiatric illness	14 (35)
Depression: Beck Depression Inventory-II, n (%)	
BDI 0-19 (none, minimal or mild)	19 (48)
BDI 20-28 (moderate)	5 (12)
BDI 29-63 (severe)	16 (40)
**Drug and Alcohol Use**	
Alcohol (by AUDIT), n (%)	
At risk alcohol (AUDIT ≥ 8)	3 (8)
Not at risk alcohol (AUDIT < 8)	37 (92)
Self-reported illicit drug use - past 30 days, n (%)	
Cocaine/crack within 30 days	9 (23)
Heroin within 30 days	9 (23)
Any heroin/cocaine/crack within 30 days	15 (38)
Years of methadone maintenance, median (IQR)	10 (5 - 18)
Median methadone dose - mg, median (IQR)	90 (60 - 140)
**HCV-related Factors**	
HCV genotype, n (%)	
Genotype 1 or 4	24 (60)
Genotype 2	9 (23)
Genotype 3	7 (17)
HCV Viral Load (IU/ml), n (%)	
≥ 800,000 IU/ml	15 (38)
< 800,000 IU/ml	25 (62)
HIV status, n (%)	
HIV-	29 (73)
HIV+	11 (27)
CD4 (cells/mm3), median (IQR)	476 (356 - 820)
Taking HAART, n (%)	7 (64)

a missing data

b depression, anxiety disorder, psychotic disorder, post-traumatic stress disorder, and/or bipolar disorder

The median estimated duration of HCV infection is 10 years (IQR 5 - 18), and 8% of participants had received HCV treatment before. Overall, 60% have genotypes 1 or 4, 23% have genotype 2, and 17% have genotype 3. Among the 11 HIV-infected subjects, 73% have undetectable HIV viral load, the median CD4+ T-cell count is 476 cells/mm^3 ^(IQR 356 - 820), and 64% are on antiretroviral therapy.

Our intervention is feasible and can be implemented in a busy clinical setting. Staff at the DoSA clinics worked collaboratively with the study team, and to date 40 participants have been enrolled from 6 out of 9 clinics. Health care providers communicate well with the Medical Director and research assistants, and nurses at each clinic record daily events on study calendars.

The DOT program is acceptable to our patients. To date, 97% of all patients who were eligible for receiving HCV treatment and met study inclusion and exclusion criteria have chosen to participate. No participants have refused their randomization.

The core components of our DOT program do not require additional funding over existing clinic operating budgets. We do not provide salary support for medical providers, nurses, or support group facilitators. HCV medications are purchased through patients' existing insurance plans, as patients would have initiated HCV treatment even if not enrolled in our study. We do supply pillboxes used for DOT, and we pay the DoSA central pharmacist a stipend to assist with management of subjects in the DOT arm.

## Discussion

To our knowledge, this study represents the first randomized controlled trial of a directly observed HCV program in a methadone program that focused specifically on the contribution of directly observed ribavirin.

A recent randomized trial of HCV treatment in methadone-maintained patients focused on directly observed pegylated interferon, and compared weekly provider-administered (DOT) pegylated interferon alfa-2a in combination with self-administered ribavirin to self-administered pegylated interferon alfa-2a in combination with self-administered ribavirin. Although subjects in both arms took ribavirin on their own, more subjects in the DOT pegylated interferon group were >97% adherent with planned cumulative doses of both peginterferon alfa-2a and ribavirin, as well as with the prescribed duration of treatment [10]. An important limitation to this trial, however, is that ribavirin adherence was assessed by patient diary rather than by more objective methods such as pill counts. Our trial builds on this by investigating the additional benefit of extending the DOT model to ribavirin, and by using an objective measure of ribavirin adherence.

Some studies have suggested that ribavirin exposure may be particularly important in determining response to HCV treatment, supporting the concept that adherence to daily ribavirin requires unique interventions. One study showed that reducing pegylated interferon from 80% to less than 60% did not have an impact, but reducing ribavirin from 80% to less than 60% was associated with significant decline in sustained viral response [7]. Another study demonstrated that adherence to combination treatment may be critically important during the first 12 weeks of treatment, and significant reductions in either drug, but especially ribavirin, reduced response to HCV treatment [18]. Both of these studies were based on provider-initiated dose reductions, and did not take into account adherence when calculating ribavirin exposure. However, measures of ribavirin exposure must include provider-initiated dose modifications, treatment interruptions, and adherence [24]. In our trial, we are measuring all of these components.

To date, our trial has enrolled a largely minority population (89%), including both Latinos and African Americans. SVR rates have been shown to be lower in Latinos and African Americans in prior studies [27-29]. Because our substance abuse treatment program serves predominantly Latino and African-American patients, we are able to study our intervention with a population known to have lower HCV treatment responses. In addition, prior studies have found that active regular drug use (defined as daily or every other day drug use) is associated with poor adherence in methadone-maintained drug users undergoing HCV treatment [2,3]. Our trial will allow us to determine whether this association will remain true for drug users initiating directly observed HCV treatment.

Despite its strengths, trial limitations should be noted. In our mDOT intervention, only some doses of ribavirin are observed. However, in one study of directly observed antiretroviral therapy among HIV-infected subjects, the percent of observed doses was not associated with virologic failure [30], suggesting that even minimal participation in a DOT program may improve antiretroviral adherence. In addition, some aspects of our study design may improve adherence in the TAU arm, and therefore reduce our measured effect size. These include weekly provider-administered pegylated interferon, frequent visits with research assistants, and participation with HCV support groups. To minimize a differential effect between groups, we balanced the two study arms regarding the amount of exposure to research staff, and we refer all subjects in both arms to HCV support groups. We also measure the degree of involvement with on-site support groups for all study subjects at each monthly study visit.

We expect that results of our HCV DOT trial will advance our knowledge of the efficacy of directly observed therapy for HCV-infected methadone maintained drug users, and inform the delivery of HCV care in methadone maintenance treatment programs. If our results provide evidence that DOT programs are effective in methadone programs, future research protocols can be developed to implement and evaluate DOT programs in other types of substance abuse treatment programs, HIV clinics, and community health centers. Data from this study will also allow us to examine the optimal degree of adherence necessary to achieve treatment success in methadone-maintained drug users undergoing HCV treatment, and will inform an effect size for larger trials. With newer regimens for HCV treatment emerging (e.g. telaprevir and boceprevir) that involve multiple oral agents in combination with pegylated interferon along with the potential for inducing resistance, DOT strategies may have even greater clinical benefit [31,32].

## Competing interests

AHL has previously served as a speaker for Roche Pharmaceuticals.

## Authors' contributions

AHL obtained funding for the study, oversaw the study's conduct, developed and implemented the study protocols, oversaw research assistants, and wrote the initial and final drafts of the manuscript. KMB developed the study protocols and provided guidance on study design and methods. XL managed study data and conducted power analyses and planned statistical analyses. JH assisted in the development of study protocols, and collected and managed study data. JHA obtained funding for the study, provided guidance on study design, methods and protocols, and completed the final manuscript version. All authors have read, contributed to, and approved the manuscript.

## Pre-publication history

The pre-publication history for this paper can be accessed here:

http://www.biomedcentral.com/1471-2334/11/315/prepub
